# Anabolic-androgenic steroid administration increases self-reported aggression in healthy males: a systematic review and meta-analysis of experimental studies

**DOI:** 10.1007/s00213-021-05818-7

**Published:** 2021-03-20

**Authors:** Razieh Chegeni, Ståle Pallesen, Jim McVeigh, Dominic Sagoe

**Affiliations:** 1 Department of Psychosocial Science, University of Bergen, Christiesgate 12, 5015 Bergen, Norway; 2 Optentia, the Vaal Triangle Campus of the North-West University, Vanderbijlpark, South Africa; 3 Department of Sociology, Manchester Metropolitan University, Manchester, UK

**Keywords:** Anabolic-androgenic steroids, Aggression, Meta-analysis, Randomized controlled trial

## Abstract

**Rationale:**

Aggression and irritability are notable psychiatric side effects of anabolic-androgenic steroid (AAS) use. However, no previous study has systematically reviewed and quantitatively synthesized effects reported by experimental studies on this topic.

**Objective:**

We conducted a systematic review and meta-analysis of randomized controlled trials (RCTs) investigating the effect of AAS administration on self-reported and observer-reported aggression.

**Methods:**

Twelve RCTs comprising a total of 562 healthy males were identified through systematic searches of MEDLINE, PsycInfo, ISI Web of Science, ProQuest, Google Scholar, and the Cochrane Library.

**Results:**

After excluding one outlier, AAS administration was associated with an increase in self-reported aggression under a random-effects model, albeit small (Hedges’ *g* = 0.171, 95% CI: 0.029–0.312, *k* = 11, *p* = .018), and when restricting the analysis to the effect of acute AAS administration on self-reported aggression under a fixed-effect model (*g* = 0.291, 95% CI: 0.014–0.524, *p* = .014). However, the above effects were neither replicated in the analysis of observer-reported aggression nor after restricting the analysis to the effects of the administration of higher (over 500 mg) and long-term (3 days to 14 weeks) doses.

**Conclusions:**

The present meta-analysis provides evidence of an increase, although small, in self-reported aggression in healthy males following AAS administration in RCTs. Ecologically rational RCTs are warranted to better explore the effect of AAS administration on aggression in humans.

**Supplementary Information:**

The online version contains supplementary material available at 10.1007/s00213-021-05818-7.

## Introduction

Anabolic-androgenic steroids (AAS) are a family of hormones comprising the androgen hormone testosterone as well as its synthetic derivatives (Kanayama and Pope 2018). Use of AAS was historically associated with weightlifters and later with professional bodybuilders and elite athletes in various sports. Since the 1980s, use of AAS has gradually spread to recreational athletes as well as the general population (Pope and Kanayama 2012). Use of AAS normally comprises long-term administration of supraphysiological doses often 10–100 times the natural production or therapeutic doses of androgens (Kanayama et al. 2013). A meta-analysis on the global prevalence of AAS use indicated that 3.3% of the world’s population has used AAS at least once with use being more frequent among males (6.4%) compared to (1.6%) females (Sagoe et al. 2014b; Sagoe and Pallesen 2018).

Despite benefits such as increased muscle growth, improved body image, and enhanced sports performance (Evans 2004; Sagoe et al. 2014a; Smit et al. 2020a), human case studies, surveys, and experimental studies suggest that AAS induce a plethora of physical and psychological adverse side effects. Cardiovascular disorders, particularly cardiomyopathy, are major physical side effects of AAS use (Baggish et al. 2017). Other somatic side effects of AAS include hypertension, sleep abnormalities, immunological dysregulation, decreased libido in males, and hirsutism and clitoromegaly in females (Bensoussan and Anderson 2019; Ganesan et al. 2020). Notable psychological side effects comprise manic and depressive symptoms as well as psychotic symptoms (Brower 2009; Kanayama et al. 2020). Human case studies, surveys, and experimental studies further suggest that AAS induce a plethora of symptoms such as irritability and unprovoked aggression sometimes referred to as “roid rage” or “steroid rage” (Nelson 1989; Pope and Katz 1987; Taylor 1987; Tragger 1988). Experimental animal studies show consistently that injections of AAS increase aggression (Clark and Henderson 2003; Lumia et al. 1994). For human studies, cross-sectional (Ganson and Cadet 2019; Pereira et al. 2019), case-control (Klötz et al. 2007; Lundholm et al. 2010; Thiblin et al. 2015), and longitudinal (Beaver et al. 2008) researches indicate a positive relationship between AAS use and aggression. However, results from human placebo-controlled randomized studies show an inconsistent association between AAS administration and aggression comprising negative (Björkqvist et al. 1994), positive (Panagiotidis et al. 2017; Wagels et al. 2018), and non-significant findings (Tricker et al. 1996).

Most previous reviews on this topic are merely narrative (Haug et al. 2004; Huo et al. 2016; Johnson et al. 2013). Additionally, a recent review (Geniole et al. 2020) on this topic lacks some studies (Anderson et al. 1992; Björkqvist et al. 1994; Su et al. 1993; Tricker et al. 1996). Hence, a comprehensive systematic review quantifying findings on the topic is overdue in line with the merit of meta-analyses in science and evidence-based medicine (Murad et al. 2016). Against this backdrop, we conducted a systematic review and meta-analysis of randomized controlled trials (RCTs) examining the effect of AAS administration on self-reported as well as observer-reported aggression in healthy males.

## Methods

### Literature search strategy

**Fig. 1:**
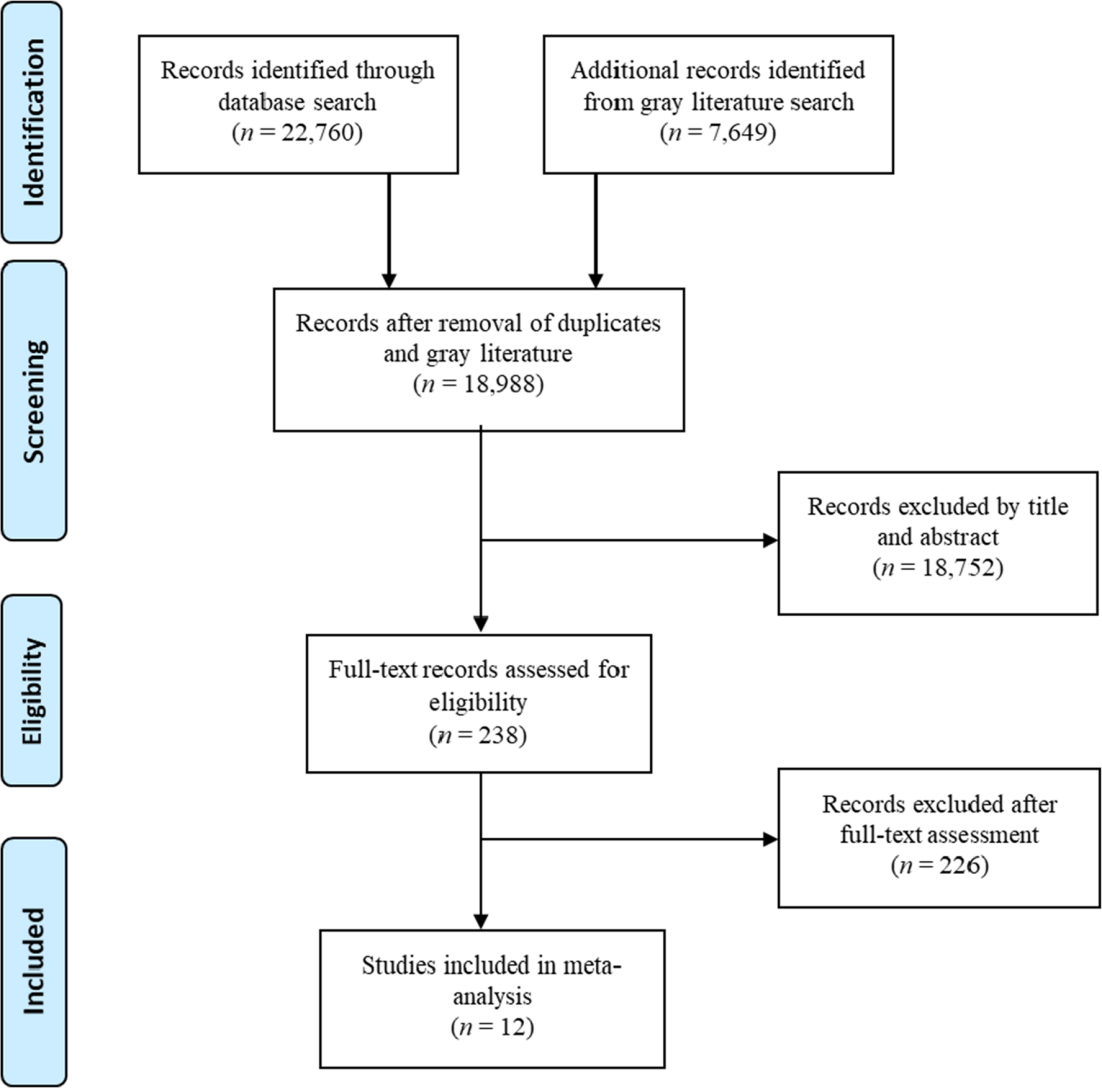
PRISMA-style flow diagram of the study selection process

Systematic literature searches were conducted in MEDLINE, PsycInfo, ISI Web of Science, ProQuest, Google Scholar, and Cochrane Library. There was no time constraint for the search. Keywords for AAS were combined with keywords for aggression. An overview of the keywords and search strategy can be found in Appendix A in the Supplementary information. The latest systematic literature search was conducted on 31 December 2019 followed by additional ad hoc searches to ensure comprehensiveness. The search and selection process are presented in Fig. 1.

### Inclusion criteria and data extraction

Included studies were as follows: (1) RCTs, (2) investigating the effects of AAS administration on aggression in healthy persons, (3) based on valid aggression measures, and (4) published in English. The first author (RC) independently conducted the search and selection of articles based on the aforementioned criteria. Using a standardized data extraction form, the first and last (RC and DS) authors independently extracted the following data from the identified studies: study authors, country, design (e.g., double-blind), sample type (e.g., healthy males), sample size, age (range, *M* ± *SD*), study groups (e.g., placebo group), AAS type, AAS dose, AAS administration mode (e.g., injection), study duration, assessment type (e.g., self-report), aggression measure, results, and risk of bias (see Table 1). Furthermore, the testosterone levels both at baseline and post-administration for each study are shown in Table 2. The two authors reached consensus in cases of discrepant extractions through discussions, with the involvement of the second author SP) when necessary. We also contacted corresponding authors or, when unavailable, coauthors via email for missing information.

**Table 1:** Characteristics of randomized controlled trials on the effects of AAS administration on aggression in healthy persons

1st author year	Country	Design	Sample type	*N* (D/E)	Age range (*M±SD*)	Groups	AAS	AAS dose	AAS mode	Study duration	Aggression measure	Results
Anderson 1992	UK	Single-blind, PL-controlled, crossover	Healthy males	31	21–41	1: Baseline (*n* = 31) 1.2: TE (*n* = 16) 1.3: PL/TE (*n* = 15)	TE	1: Baseline 1.2: TE–200 mg TE–1× × 8wks 1.3: PL/TE–PL 1× × 4wks ffd by 200 mg TE 1× × 4wks	Injection	16wks	Daily ratings (irritable, ready to fight, easily angered)	No dose effect (*p*s > .05)
Björkqvist 1994	Finland	Double-blind, PL-controlled	Healthy males	27 (3)	21–31 (23.9±)	1: TU (*n* = 9) pc 2: PL (*n* = 9) pc 3: Control (*n* = 9) pc	TU (Panteston®)	40 mg × 1×	Oral	≈4.3wks	VAS (SEMC) (irritability, anger)	PL > TU, and control (*p*s < .02)
Carré 2017	Canada	Double-blind, PL-controlled	Healthy males	114 (7)	18–35 (25.27±4.98)	1: TG (*n* = 57) 2: PL (*n* = 57)	TG (AndroGel®)	150 mg (pc) × 1×	Transdermal (gel)	1×	PSAP	TG vs PL: No dose effect (*p* = .11) BSC × (TG vs PL): dose effects (ps < .02)
Cueva 2017	UK	Double-blind, PL-controlled, crossover	Healthy males	38	22.4±2.97	1: Baseline (*n* = 20) 1.2: TE (*n* = 20) 1.3: PL/TE (*n* = 18)	T (Testogel™)	100 mg 3× × 3d pc	Transdermal (gel)	3 days	VAS (aggression)	No dose effect (*p* = .06)
Dreher 2016	Ireland	Double-blind, PL-controlled	Healthy males	40	18–30 (21.25±2.97)	1: TU (*n* = 21) pc 2: PL (*n* = 19) pc	TE	250 mg	Injection	1×	POMS (anger)	No dose effect (*p* = .43)
O’Connor 2002	UK	Double-blind, PL-controlled	Healthy males	30	19–45 (28.2±)	1: TE wks 0–4–8 (*n* = 15) 2: PL wks 0–4–8 (*n* = 15)	TE	200 mg 1×/wk × 8wks	Injection	8wks	POMS (anger-hostility) BPAQ APQ BDHI (irritability) BIS-11	No dose effect (*p*s > .05)
O’Connor 2004	UK	Double-blind, PL-controlled, crossover	Healthy males	24 (4)	22–44 (32.29±6.13)	1: TU (*n* = 13) pc 2: PL *(n* = 11) pc	TU	1000 mg 1×	Injection	28wks	POMS (anger-hostility) BPAQ APQ BDHI (irritability)	POMS (anger-hostility): TU wk2 > TU wk0 (*p* < .05) No other dose effect (*p*s > .05)
Panagiotidis 2017	Germany	Double-blind, PL-controlled	Healthy males	83 (7)	18–35	1: TG (*n* = 42; age range 18–35, 24.45±3.78) 2: PL (*n* = 41; age range 18–31, 23.89±3.65)	TG (Testim®)	50 mg × 1×	Transdermal (gel)	1×	TPP ESR (anger)	TPP × ESR: TG > PL (FAIL condition, *p* = .041); TG vs PL (GO condition, *p* = .72) TG × ESR: *T*_FAIL_ > T_GO_ (*p* < .001)
Pope 2000	USA	Double-blind, PL-controlled, crossover	Healthy males	53 (T1: 3, T2: 6)	25–49	1: TC (*n* = 23) 2: PL (30)	TC	1: 150 mg 1×/wk × 2wks 2: 300 mg 1×/wk × 2wks 3: 600 mg 1×/wk × 2 wks	Injection	25wks	PSAP (*n* = 27) BPAQ	PSAP: TC > PL (*p* = .03) BPAQ: No dose effect (*p* = .35)
Su 1993	USA	Double-blind, PL-controlled, crossover	Healthy males	20 (3)	18–42 (27.5±5.7)	1: Baseline (*n* = 20) 2: MT 40 mg (*n* = 20) 3: MT 240 mg (*n* = 20) 4: Withdrawal (*n* = 20)	MT	2: 40 mg–3×/d × 3d 3: 240 mg–3×/d × 3d	Oral	2wks	VAS (irritability, anger, violent feelings) SCL-90 (hostility)	Irritability: MT 240 mg > Baseline (*p* < .05) No other dose effect (*p*s > .05)
Tricker 1996	USA	Double-blind, PL-controlled	Healthy males	40 (3)	19–40	1: PL only (*n* = 10; age: 27±5) 2: TE only (*n* = 10; age: 26±6) 3: PL × ST 3×/wk (*n* = 9; age: 26±6) 4: TE × ST 3×/wk (*n* = 11; age: 30±7)	TE	600 mg 1×/wk × 10wks	Injection	30wks	MAI	No dose effect: anger arousal, anger situations, hostile outlook, anger-in, anger-out (*p*s > .05)
Yates 1999	USA	Double-blind, PL-controlled, crossover	Healthy males	32 (11)	21–40	1: PL (*n* = 32) 1.2: TC 100 (*n* = 10; age: 27.4±3.3) 1.3: TC 250 (*n* = 11; age: 27.5±5.5) 1.4: TC 500 (*n* = 11; age: 30.2±5.9)	TC	1.2: 100 mg 1×/wk × 14wks 1.3 250 mg 1×/wk × 14wks 1.4: 500 mg 1×/wk × 14wks	Injection	28wks	BDHI (assault)	No dose effect (*p* = .79)

1×: 1 time. *APQ*, Aggressive Provocation Questionnaire; *BDHI*, Buss-Durkee Hostility Inventory; *BPAQ*, Buss-Perry Aggression Questionnaire; *BPAQ-P*, Buss-Perry Aggression Questionnaire-Partner; *BSC*, Brief Self-Control Scale; *D/E*, dropouts or excluded; *ESR*, Emotional Self-Ratings; *MAI*, Multi-Dimensional Anger Inventory; *MT*, methyltestosterone; *OMI*, Observer Mood Inventory; pc, Personal communication; PL, Placebo; *POMS*, Profile of Mood States; *PSAP*, Point Subtraction Aggression Paradigm; *SCL-90*, Symptom Checklist 90; *SEMC*, Self-Estimated Mood Checklist; *ST*, strength training; *T*, testosterone; *TC*, testosterone cypionate; *TE*, testosterone enanthate; *TG*, testosterone gel; *TU*, testosterone undecanoate; *TPP*, Technical Provocation Paradigm; *VAS*, visual analogue scale

**Table 2:** Mean baseline and post-administration levels of placebo and testosterone for each study (nmol/L)

1st author year	Placebo		Testosterone	
	Baseline	Post-administration	Baseline	Post-administration
Anderson 1992	19.20	33.10	17.70	28.80
Björkqvist 1994	-	-	-	-
Carré 2017	18.38	19.07	19.07	30.16
Cueva 2017	1.04	1.04	.69	10.05
Dreher 2016	20.46	20.44	21.06	66.08
O’Connor 2002	20.10	20.0	21.70	38.42
O’Connor 2004	20.30	20.30	20.70	37.50
Panagiotidis 2017	16.99	15.0	16.62	21.20
Pope 2000	16.30	18.40	17.40	76.00
Su 1993	-	-	-	-
Tricker 1996	18.60	19.40	16.10	76.90
Yates 1999	20.82	19.08	20.82	73.73

### Statistical analysis

We first investigated the overall effect of AAS administration on self-reported aggression using a random-effects model. AAS users typically administer supraphysiologic doses of AAS for 4 to 28 weeks (Kanayama et al. 2013; Copeland et al. 2000). We therefore subsequently pooled studies in which higher doses (over 500 mg) of AAS were administered for the examination of the effect of high-dose AAS administration on self-reported aggression (O’Connor et al. 2004; Pope et al. 2000; Su et al. 1993; Tricker et al. 1996; Yates et al. 1999). Furthermore, we pooled studies in which AAS were administered over longer periods (i.e., 3 days to 14 weeks: Anderson et al. 1992; Cueva et al. 2017; O’Connor et al. 2002; O’Connor et al. 2004; Pope et al. 2000; Su et al. 1993; Yates et al. 1999) as well as studies investigating acute AAS effects (Carré et al. 2017; Dreher et al. 2016; Panagiotidis et al. 2017; Tricker et al. 1996). Due to the low number of studies administering higher doses (*k* = 5) or investigating acute AAS effects (*k* = 4), a fixed-effect model was used for these analyses (Borenstein 2009). Moreover, we conducted a meta-regression analysis to elucidate a potential dose-response association, regressing AAS dose (mg) on self-reported aggression. Finally, we investigated the overall effect of AAS administration on observer-reported aggression using a fixed-effect model due to the low number of studies (*k* = 3: O’Connor et al. 2004; Tricker et al. 1996; Yates et al. 1999).

Some studies used multiple aggression measures and reported multiple aggression scores (O’Connor et al. 2002, 2004; Panagiotidis et al. 2017; Pope et al. 2000; Su et al. 1993). In these cases, we set the correlation between aggression measures to 0.60 (Diamond and Magaletta 2006; O’Connor et al. 2001) to provide the best estimates of between-study variance and corresponding confidence intervals (Gleser and Olkin 2009; Marín-Martínez and Sánchez-Meca 1999). For crossover studies (O’Connor et al. 2004; Pope et al. 2000; Su et al. 1993; Yates et al. 1999), we used an average correlation of 0.50 between aggression measures over time to provide optimal effect size estimates (Krahé and Möller 2010). Effects were estimated as Hedges’ *g*, where 0.20 is considered small, 0.50 moderate, and 0.80 as large effect sizes, respectively (Hedges and Olkin 2014). For studies including a passive control group (e.g., no intervention), a placebo group, and a treatment group (Björkqvist et al. 1994), data from the placebo and treatment groups were used to estimate meaningful relative-effect estimates (Karlsson and Bergmark 2015; Magill and Longabaugh 2013). Effect sizes were calculated by pooling post-intervention mean and standard deviations of aggression scores. When mean and standard deviation were not reported or unavailable in the original paper, authors were approached by email (Björkqvist et al. 1994), and asked to provide statistical information (i.e., *F* and *p* values) necessary to calculate effect sizes. For the assessment of heterogeneity, we used the *Q*-statistic and the *I*^2^ index. The latter indicates the proportion of the observed variance that reflects real differences in effect size. It is expressed as a percentage (0–100) with 0% indicating no heterogeneity, 25% indicating low heterogeneity, 50% indicating moderate heterogeneity, and 75% suggesting high heterogeneity (Higgins et al. 2003) respectively. Additionally, we used Duval and Tweedie’s (2000) trim and fill method, and Orwin’s (1983) fail-safe *N* to assess publication bias. The trim and fill method (Duval and Tweedie 2000) screens for missing studies and adjusts the effect size by trimming the asymmetric studies and filling a funnel plot symmetrically. Orwin’s (1983) fail-safe *N* quantifies the number of studies required to bring the observed effect size down to a chosen “trivial” estimate (Hedges and Olkin 2014). In the current meta-analysis, we set the “trivial” estimate to *g* of 0.05.

The quality of each included study was assessed using the Cochrane risk of bias tool (Higgins et al. 2003). The protocol for the meta-analysis was pre-registered in PROSPERO (CRD 42019117834). The literature search, coding of variables, and reporting were conducted according to the Preferred Reporting Items for Systematic Reviews and Meta-Analyses (PRISMA) procedure (Moher et al. 2009). The meta-analysis and the meta-regression were performed using the Comprehensive Meta-Analysis version 3.3.070 (Borenstein et al. 2014).

## Results

### Literature screening and selection

From an initial pool of 30,407 hits, 18,988 records remained after removal of duplicates (*k* = 3772) and gray literature (*k* = 7649) during initial identification and screening. Of this pool, 18,752 were removed after eligibility screening by title and abstract leaving 238 records for further evaluation. After screening the 238 full-text records, 12 studies were finally included. Figure 1 presents the literature search and selection process.

### Description of included studies

Of the twelve included studies, publication year ranged from 1992 (Anderson et al. 1992) to 2017 (Carré et al. 2017; Cueva et al. 2017; Panagiotidis et al. 2017). Four of the studies were conducted in the USA (Pope et al. 2000; Su et al. 1993; Tricker et al. 1996; Yates et al. 1999), four in the UK (Anderson et al. 1992; Cueva et al. 2017; O’Connor et al. 2002, 2004), and one each in Germany (Panagiotidis et al. 2017), Finland (Björkqvist et al. 1994), Ireland (Dreher et al. 2016), and Canada (Carré et al. 2017). We received clarification and data from some authors (Björkqvist et al. 1994; Carré et al. 2017; Cueva et al. 2017; Dreher et al. 2016; O’Connor et al. 2004). (See Table 1.)

All the included studies comprised placebo-controlled randomized trials. One of the included studies was single-blinded (Anderson et al. 1992) and 11 were double-blinded. Additionally, six studies were crossover studies (Anderson et al. 1992; Cueva et al. 2017; O’Connor et al. 2004; Pope et al. 2000; Su et al. 1993; Yates et al. 1999) whereas five were based on a between-subject design (Björkqvist et al. 1994; Carré et al. 2017; Dreher et al. 2016; O’Connor et al. 2002; Panagiotidis et al. 2017; Tricker et al. 1996). The studies included a total of 562 healthy male (females: *n* = 0) participants. Participants’ ages ranged from 18 (Su et al. 1993) to 49 (Carré et al. 2017) with a grand mean of 25.83 (*SD* = 3.80).

Testosterone enanthate was administered in four studies (Anderson et al. 1992; Dreher et al. 2016; O’Connor et al. 2002; Tricker et al. 1996) and two studies administered testosterone cypionate (Pope et al. 2000; Yates et al. 1999). In addition, two studies administered testosterone undecanoate (Björkqvist et al. 1994; O’Connor et al. 2004), and three studies administered testosterone gel (Carré et al. 2017; Cueva et al. 2017; Panagiotidis et al. 2017) whereas one study administered methyltestosterone (Su et al. 1993). AAS doses ranged from a one-time application of 50 mg of testosterone gel (Panagiotidis et al. 2017) to a one-time injection of 1000 mg of testosterone undecanoate (O’Connor et al. 2004), and a cumulative injection of 7000 mg of testosterone cypionate over a 14-week period (Yates et al. 1999). When various doses of AAS were used in one study, we used results from the highest dose for calculating the effect size.

Aggression was assessed by self-reports (Anderson et al. 1992; Björkqvist et al. 1994; Carré et al. 2017; Cueva et al. 2017; Dreher et al. 2016; O’Connor et al. 2002, 2004; Panagiotidis et al. 2017; Pope et al. 2000; Su et al. 1993; Tricker et al. 1996; Yates et al. 1999), observer-reports (O’Connor et al. 2004; Tricker et al. 1996; Su et al. 1993; Yates et al. 1999), and behavioral aggression measures (Carré et al. 2017; Pope et al. 2000). The Buss-Perry Aggression Questionnaire (Buss and Perry 1992) was used in three studies (O’Connor et al. 2002, 2004; Pope et al. 2000), and three studies (O’Connor et al. 2002, 2004; Yates et al. 1999) used the Buss-Durkee Hostility Inventory (Buss and Durkee 1957), two studies (Carré et al. 2017; Pope et al. 2000) used the Point Subtraction Aggression Paradigm (Cherek et al. 1996), and three studies (Dreher et al. 2016; O’Connor et al. 2002, 2004) used the Profile of Mood States (McNair et al. 1992) with two out of these three studies (O’Connor et al. 2002, 2004) additionally using the Aggression Provocation Questionnaire (O’Connor et al. 2001).

Additionally, the Self-Estimated Mood Checklist (Lindman 1985) was used in one study (Björkqvist et al. 1994), and one study (Panagiotidis et al. 2017) used the Technical Provocation Paradigm (Panagiotidis et al. 2017) and emotional self-ratings (Schneider et al. 1994). Moreover, two studies (Cueva et al. 2017; Su et al. 1993) used visual analogue scales (Cline et al. 1992; Norris 1971), one study (Tricker et al. 1996) used the Multi-Dimensional Anger Inventory (Siegel 1986), and one study (Anderson et al. 1992) used daily ratings of irritability, readiness to fight, and being easily angered. 10 studies (Anderson et al. 1992; Carré et al. 2017; Cueva et al. 2017; Dreher et al. 2016; O’Connor et al. 2002, 2004; Panagiotidis et al. 2017; Pope et al. 2000; Tricker et al. 1996; Yates et al. 1999) reported no significant effect of AAS administration on aggression. In addition, one study (Su et al. 1993) found a positive effect of AAS administration on aggression (*p* < .05), whereas one study (Björkqvist et al. 1994) reported a negative effect of AAS administration on aggression (*p* < .01).

### Risk of bias

**Fig. 2:**
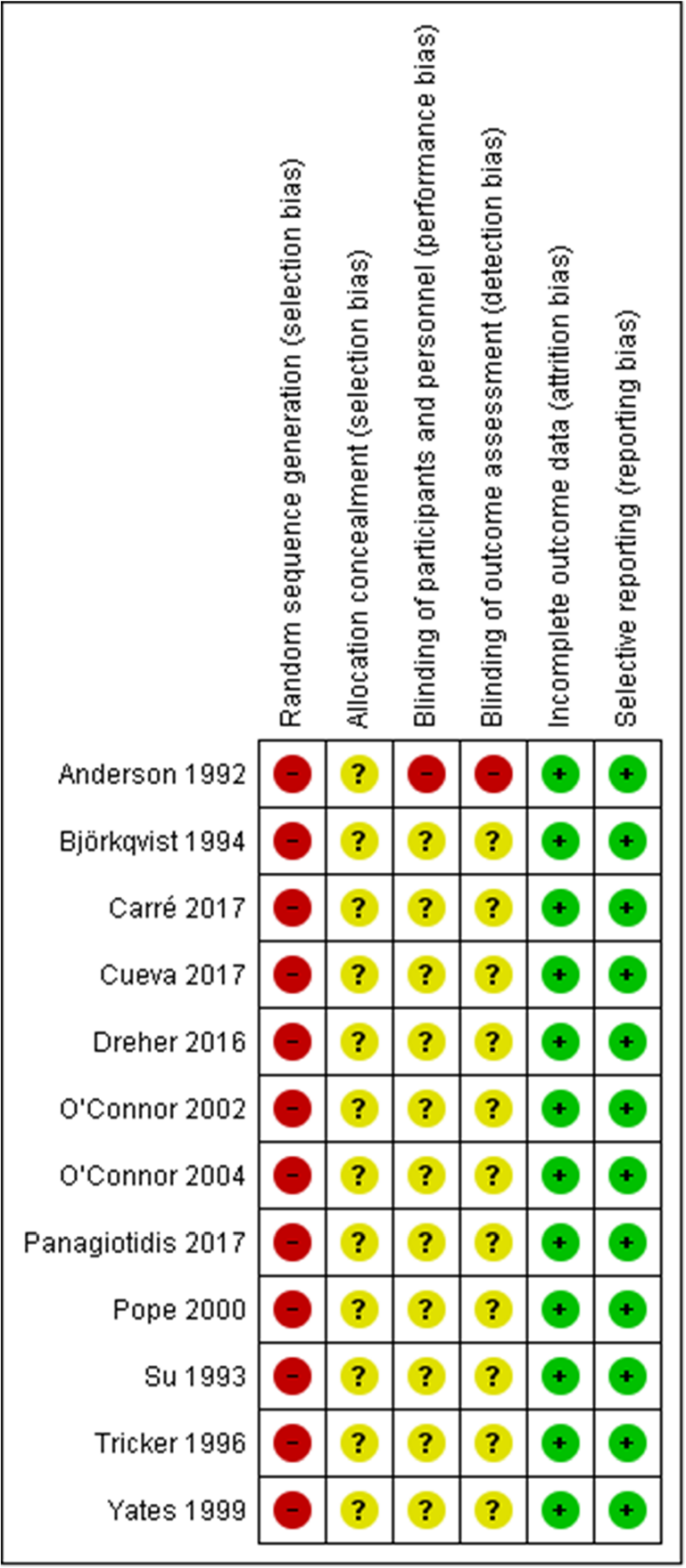
Estimated risk of bias of the included studies

The two authors disagreed once on the random sequence generation dimension for all the included studies yielding a Cohen’s kappa of .58 (Cohen 1988). All studies were evaluated as having a high selection bias as there was no description of the randomization method or concealed allocation process. In addition, all studies were evaluated as having high risks of performance and detection bias as the effectiveness of blinding was not tested. Moreover, all studies had a low risk of attrition bias as there was sufficient reporting and handling of attrition and exclusion. Furthermore, except for one study that did not present means and standard deviations or inferential indices (Björkqvist et al. 1994), we evaluated all studies as having low reporting bias. Figure 2 depicts the risk of bias of the included studies.

### Effect of AAS administration on self-reported aggression

**Fig. 3:**
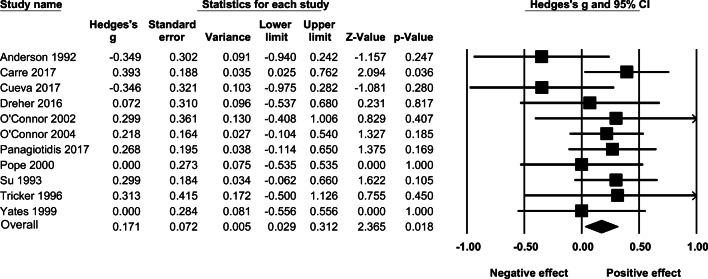
The effect (random-effects model) of AAS administration on self-reported aggression

Of the twelve included studies, one study (Björkqvist et al. 1994) did not overlap with the 95% CI of the overall pooled effect size. Exclusion of this outlier resulted in a mean and significant random-effects size of *g* = 0.171 (95% CI: 0.029–0.312, *k* = 11, *p* = .018), and there was no significant heterogeneity between the included studies (*I*^2^ = 0.000, *Q =* 8.891, *p* = .542). The effect sizes and associated 95% confidence intervals are presented in Fig. 3.

The overall random-effects of AAS administration on self-reported aggression, including the outlier (Björkqvist et al. 1994), was not significant (*g* = 0.081, 95% CI: −0.111–0.273, *p* = .408). (See Supplementary Figure 1.) When adjusting for publication bias using Duval and Tweedie’s trim and fill method, the overall result (*k* = 12) turned out non-significant (*g* = 0.170, 95% CI: 0.029–0.312, *p* = .890). (See Supplementary Figure 2.) Results from Orwin’s fail-safe *N* analysis indicated that 27 studies with an effect size of zero would be needed to bring Hedges’ *g* below 0.05.

### Effect of long-term AAS administration on self-reported aggression

**Fig. 4:**
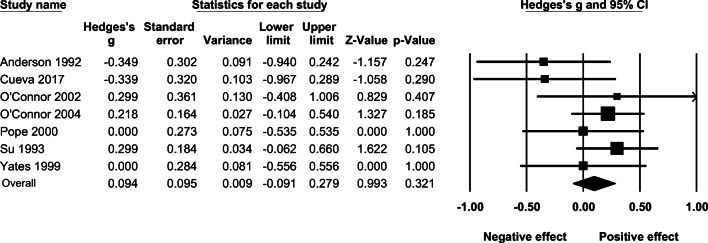
The effect (random-effects model) of administering AAS over longer periods on self-reported aggression

The random-effects of administering AAS over longer periods (3 days to 14 weeks) on self-reported aggression under a random-effects model was *g* = 0.100 (95% CI:−0.079–0.278, *p* = .273). There was no significant heterogeneity across studies in terms of effect sizes (*I*^2^ = 5.286, *Q =* 6.335, *p* = .321). (See Fig. 4.)

### Effect of acute AAS administration on self-reported aggression

**Fig. 5:**
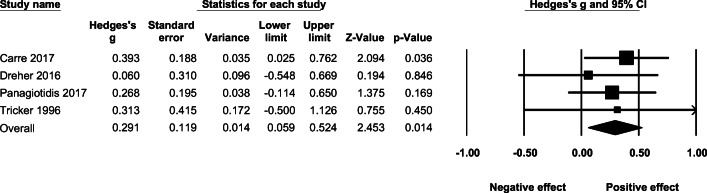
The effect (fixed-effect model) of acute AAS administration on self-reported aggression

Under a fixed-effect model, the effect of acute administration of AAS on self-reported aggression was *g* = 0.291 (95% CI: 0.014–0.524, *p* = .014, *Q* =.867, *p* = .833 ). (See Fig. 5.)

### Effect of AAS dose on self-reported aggression

AAS dose (mg) was not associated with self-reported aggression in a random-effects meta-regression model (*B* = 0.000, *SE* = 0.000 (95% CI: −0.000–0.000), *p* = .096).

### Effect of high-dose AAS administration on self-reported aggression

**Fig. 6:**
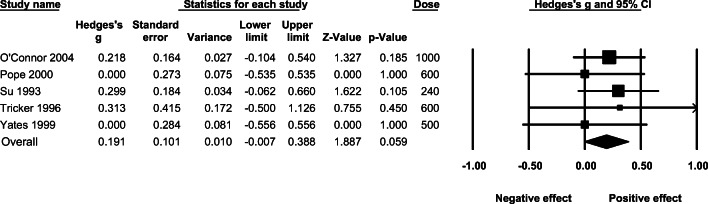
The effect (fixed-effect model) of administering higher (over 500 mg) doses of AAS on self-reported aggression

The mean effect of higher doses (over 500 mg) of AAS on self-reported aggression under a fixed-effect model was non-significant (*g* = 0.191; 95% CI: −0.007–0.388, *p* = .059, *Q* = 1.399, *p* = .844). (See Fig. 6.)

### Effect of AAS administration on observer-reported aggression

**Fig. 7:**
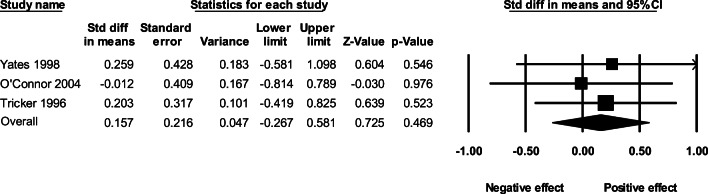
The effect (fixed-effect model) of AAS administration on observer-reported aggression

The overall fixed-effect of AAS administration on aggression based on observer ratings resulted in an effect size of *g* = 0.157 (95% CI: −0.026–0.581, *p* = .469, *Q* = .249, *p* = .833). The effect sizes and associated 95% confidence intervals for each study are presented in Fig. 7.

## Discussion

The present systematic review and meta-analysis of eleven studies (Anderson et al. 1992; Carré et al. 2017; Cueva et al. 2017; Dreher et al. 2016; O’Connor et al. 2002, 2004; Panagiotidis et al. 2017; Pope et al. 2000; Su et al. 1993; Tricker et al. 1996; Yates et al. 1999), after excluding an outlier (Björkqvist et al. 1994), indicates that AAS administration is associated with an increase in self-reported aggression, albeit small, among healthy males in RCTs. This finding is consistent with the results of a recent meta-analysis (Geniole et al. 2020) indicating that testosterone administration has a small and positive correlation with aggression in males. Relatedly, our finding that acute AAS administration has a positive effect on self-reported aggression is consistent with evidence that acute increases in testosterone have a positive correlation with aggression (Geniole et al. 2020).

The present study is the first comprehensive systematic review and meta-analytic investigation of the effect of AAS administration and aggression in healthy males in RCTs. However, our results should be interpreted with caution. Firstly, a meta-regression examining dosage as a moderator of the identified effect of AAS on self-reported aggression turned out not significant. Similarly, we did neither detect an effect of AAS administration on observer-reported aggression nor for the effects of long-term (3 days to 14 weeks) and high-dose AAS administration on self-reported aggression. Also, as noted previously, only healthy males were examined in the included RCTs and the duration and doses used in the twelve RCTs deviate from the prolonged use of high-dose cycles consisting of the ingestion of supraphysiologic doses of different types of AAS per week over several months (Kanayama et al. 2013) often reported by users in ecologically valid settings. In one study, the reported weekly AAS dose ranged from 125 to 7000 (mean = 1278) mg per week over an average of 9.1 years (Bjørnebekk et al. 2017). In another recent study, it was shown that an AAS cycle usually comprises the ingestion of five different AAS with an average dose of 901 mg per week for a typical duration of 13 weeks (Smit et al. 2020b). In the present meta-analysis, the highest dose administered was a one-time injection of 1000 mg of testosterone undecanoate (O’Connor et al. 2004) and a cumulative injection of 7000 mg of testosterone cypionate over a 14-week period (Yates et al. 1999). Inferably, AAS doses and duration of administration in the RCTs included in our meta-analysis are far lower than the actual doses reported by AAS users (Bjørnebekk et al. 2017; Kanayama et al. 2013).

Similarly, besides the administration of methyltestosterone in one study (Su et al. 1993), fluoxymesterone, oxymetholone, and trenbolone that are anecdotally associated with increased aggression in humans (Barker 1987; Llewellyn 2011) were not administered in the RCTs included in the present review. Moreover, testosterone undecanoate administered in two studies (Björkqvist et al. 1994; O’Connor et al. 2004) is a depot with a very gradual decay and long half-life leading to relatively stable testosterone levels over a prolonged period of time (Hirschhäuser et al. 1975). Hence, discrepancies in AAS doses, type, duration of use, and half-life between the AAS in the RCTs and naturalistic contexts should be noted when interpreting our findings.

In addition, evidence from cross-sectional studies indicates that polypharmacy and stacking (Sagoe et al. 2015; Salinas et al. 2019) may account for increased aggression among AAS users (Lundholm et al. 2015). The absence of polypharmacy in the RCTs included in our meta-analysis may also explain the discrepancy between findings from RCTs and those reported in more ecologically valid contexts. Other potential confounding factors include small sample sizes and lack of a priori power analyses, diversity in aggression measures, risk of bias (selection, performance, and detection biases), diversity in route of administrating AAS (injecting, transdermally), diversity in time gap between AAS administration, incomplete data reporting, and sampling of only males in included RCTs.

Moreover, the inclusion of only healthy volunteers in the RCTs may have precluded vulnerable subjects from participating which may have led to the underestimation of the effects of AAS administration on aggression. Sampling is important with evidence that testosterone increases aggression in men with certain personality profiles especially among those with fewer cytosine-adenine-guanine repeats in exon 1 of the androgen receptor gene (Geniole et al. 2019). The importance of sampling is further evidenced in that, apart from bodybuilders and competitive athletes, a large portion of non-experimental research linking AAS use with aggression has been conducted among subgroups associated with aggression such as drug users, offenders, and prisoners (Lundholm et al. 2010; Pope et al. 1996), as well as policemen, doormen, and nightclub bouncers (Hoberman 2017; Midgley et al. 2001). Future researchers considering the aforementioned factors may conduct more ecologically valid RCTs (e.g., by using dosages and duration of use similar to those by real AAS users) to better elucidate the effect of AAS administration on aggression in humans. Furthermore, more studies should explore factors of AAS administration (e.g., type of AAS, duration of use, premorbid functioning, and genetics) that might moderate the effects of AAS on aggression.

## Conclusions

The present systematic review and meta-analysis provide evidence for an increase, although small, in self-reported aggression in healthy males following AAS administration in RCTs. Moreover, when restricting the analysis to the effects of acute AAS administration on self-reported aggression, we found a significant effect. We also identified important limitations of the RCTs on issues such as non-ecological doses, lack of personality and polypharmacy controls, small sample sizes, risk of bias, short study duration, and the inclusion of only healthy males. While future RCTs adjusting for the above factors may contribute better to contemporary understanding of the effect of AAS administration on aggression in humans, the present study provides an important foundation for addressing this important public health issue. As the appreciation of the heterogeneity of AAS use matures, there is a need to identify the role that AAS plays in aggression and violence and what may be attributed to the set and setting of their use.

## Supplementary information

ESM 1 (DOCX 75 kb)

## References

[CR1] *Anderson RA, Bancroft J, Wu FC (1992) The effects of exogenous testosterone on sexuality and mood of normal men. J Clin Endocrinol Metab 75:1503–1507 10.1210/jcem.75.6.1464655 1464655

[CR2] Baggish AL, Weiner RB, Kanayama G, Hudson JI, Lu MT, Hoffmann U, Pope HG (2017). Cardiovascular toxicity of illicit anabolic-androgenic steroid use. Circulation.

[CR3] Barker S (1987). Oxymethalone and aggression. Br J Psychiatry.

[CR4] Beaver KM, Vaughn MG, DeLisi M, Wright JP (2008). Anabolic-androgenic steroid use and involvement in violent behavior in a nationally representative sample of young adult males in the United States. Am J Public Health.

[CR5] Bensoussan Y, Anderson J (2019). Case report: The long-term effects of anabolic steroids on the female voice over a 20-year period. Clin Case Rep.

[CR6] *Björkqvist K., Nygren T., Björklund AC, Björkqvist SE (1994) Testosterone intake and aggressiveness: real effect or anticipation? Aggress Behav 20:17-26

[CR7] Bjørnebekk A, Walhovd KB, Jørstad ML, Due-Tønnessen P, Hullstein IR, Fjell AM (2017). Structural brain imaging of long-term anabolic-androgenic steroid users and nonusing weightlifters. Biol Psychiatry.

[CR8] Borenstein M (2009). Introduction to meta-analysis.

[CR9] Borenstein M, Hedges L, Higgins J, Rothstein H (2014) Comprehensive meta-analysis version 3. Biostat, Englewood

[CR10] Brower KJ (2009). Anabolic steroid abuse and dependence in clinical practice. Phys Sportsmed.

[CR11] Buss AH, Durkee A (1957). An inventory for assessing different kinds of hostility. J Consult Psychol.

[CR12] Buss AH, Perry M (1992). The aggression questionnaire. J Pers Soc Psychol.

[CR13] *Carré JM, Geniole SN, Ortiz TL, Bird BM, Videto A, Bonin PL (2017). Exogenous testosterone rapidly increases aggressive behavior in dominant and impulsive men. Biol Psychiatry 82:249-256. 10.1016/j.biopsych.2016.06.009 27524498

[CR14] Cherek DR, Schnapp W, Moeller FG, Dougherty DM (1996). Laboratory measures of aggressive responding in male parolees with violent and nonviolent histories. Aggress Behav.

[CR15] Clark AS, Henderson LP (2003). Behavioral and physiological responses to anabolic-androgenic steroids. Neurosci Biobehav Rev.

[CR16] Cline ME, Herman J, Shaw ER, Morton RD (1992). Standardization of the visual analogue scale. Nurs Res.

[CR17] Cohen J (1988). Statistical power analysis for the behaviors science.

[CR18] Copeland J, Peters R, Dillon P (2000). Anabolic-androgenic steroid use disorders among a sample of Australian competitive and recreational users. Drug Alcohol Depend.

[CR19] *Cueva C, Roberts RE, Spencer TJ, Rani N, Tempest M, Tobler PN, Rustichini A (2017). Testosterone administration does not affect men’s rejections of low ultimatum game offers or aggressive mood. Hormones and Behavior 87:1-7. 10.1016/j.yhbeh.2016.09.012 27712924

[CR20] Diamond PM, Magaletta PR (2006). The short-form Buss-Perry Aggression questionnaire (BPAQ-SF) a validation study with federal offenders. Assessment.

[CR21] *Dreher JC, Dunne S, Pazderska A, Frodl T, Nolan JJ, O’Doherty JP. (2016) Testosterone causes both prosocial and antisocial status-enhancing behaviors in human males. Proc Natl Acad Sci 113:11633-11638. 10.1073/pnas.1608085113 PMC5068300 27671627

[CR22] Duval S, Tweedie R (2000). Trim and fill: a simple funnel plot–based method of testing and adjusting for publication bias in meta-analysis. Biometrics.

[CR23] Evans NA (2004). Current concepts in anabolic-androgenic steroids. Ame J Sports Med.

[CR24] Ganson KT, Cadet TJ (2019). Exploring anabolic-androgenic steroid use and teen dating violence among adolescent males. Subst Use Misuse.

[CR25] Ganesan K, Haque IU, Zito PM (2020) Anabolic Steroids. StatPearls, Treasure Island

[CR26] Geniole SN, Procyshyn TL, Marley N, Ortiz TL, Bird BM, Marcellus AL, Carré JM (2019). Using a psychopharmacogenetic approach to identify the pathways through which—and the people for whom—testosterone promotes aggression. Psychol Sci.

[CR27] Geniole SN, Bird BM, McVittie JS, Purcell RB, Archer J, Carré JM (2020). Is testosterone linked to human aggression? A meta-analytic examination of the relationship between baseline, dynamic, and manipulated testosterone on human aggression. Horm Behav.

[CR28] Gleser LJ, Olkin I, Cooper H, Hedges LV, Valentine JC (2009). Stochastically dependent effect sizes. *The handbook of research synthesis and meta-analysis*.

[CR29] Haug E, Mørland J, Olaisen B, Myhre KI (2004). Androgene-anabole steroider (AAS) og vold [Androgenic-anabolic steroids (AAS) and violence].

[CR30] Hedges LV, Olkin I (2014). Statistical methods for meta-analysis.

[CR31] Higgins JP, Thompson SG, Deeks JJ, Altman DG (2003). Measuring inconsistency in meta-analyses. BMJ.

[CR32] Hirschhäuser C, Hopkinson CRN, Sturm G, Coert A (1975). Testosterone undecanoate: a new orally active androgen. Eur J Endocrinol.

[CR33] Hoberman JM (2017). The hidden world of police on steroids.

[CR34] Huo S, Scialli AR, McGarvey S, Hill E, Tügertimur B, Hogenmiller A et al (2016) Treatment of men for “low testosterone”: a systematic review. PLoS One 11:e0162480 10.1371/journal.pone.0162480 PMC5031462 27655114

[CR35] Johnson JM, Nachtigall LB, Stern TA (2013). The effect of testosterone levels on mood in men: a review. Psychosomatics.

[CR36] Kanayama G, Pope HG (2018). History and epidemiology of anabolic androgens in athletes and non-athletes. Mol Cell Endocrinol.

[CR37] Kanayama G, Kean J, Hudson JI, Pope HG (2013). Cognitive deficits in long-term anabolic-androgenic steroid users. Drug Alcohol Depend.

[CR38] Kanayama G, Hudson JI, Pope HG (2020). Anabolic-Androgenic Steroid Use and Body Image in Men: A Growing Concern for Clinicians. Psychother Psychosom.

[CR39] Karlsson P, Bergmark A (2015). Compared with what? An analysis of control-group types in Cochrane and Campbell reviews of psychosocial treatment efficacy with substance use disorders. Addiction.

[CR40] Klötz F, Petersson A, Isacson D, Thiblin I (2007). Violent crime and substance abuse: a medico-legal comparison between deceased users of anabolic androgenic steroids and abusers of illicit drugs. Forensic Sci Int.

[CR41] Krahé B, Möller I (2010). Longitudinal effects of media violence on aggression and empathy among German adolescents. J Appl Dev Psychol.

[CR42] Lindman R (1985). On the direct estimation of mood change. Percept Psychophys.

[CR43] Llewellyn W (2011) Anabolics. Molecular Nutrition LLC, Jupiter

[CR44] Lumia AR, Thorner KM, McGinnis MY (1994). Effects of chronically high doses of the anabolic androgenic steroid, testosterone, on intermale aggression and sexual behavior in male rats. Physiol Behav.

[CR45] Lundholm L, Käll K, Wallin S, Thiblin I (2010). Use of anabolic androgenic steroids in substance abusers arrested for crime. Drug Alcohol Depend.

[CR46] Lundholm L, Frisell T, Lichtenstein P, Långström N (2015). Anabolic androgenic steroids and violent offending: Confounding by polysubstance abuse among 10,365 general population men. Addiction.

[CR47] Magill M, Longabaugh R (2013). Efficacy combined with specified ingredients: a new direction for empirically supported addiction treatment. Addiction.

[CR48] Marín-Martínez F, Sánchez-Meca J (1999). Averaging dependent effect sizes in meta-analysis: a cautionary note about procedures. Span J Psychol.

[CR49] McNair DM, Lorr M, Droppleman LF (1992) Revised manual for the Profile of Mood States (POMS). Educational and Industrial Testing Service, San Diego

[CR50] Midgley SJ, Heather N, Davies JB (2001). Levels of aggression among a group of anabolic-androgenic steroid users. Med Sci Law.

[CR51] Moher D, Liberati A, Tetzlaff J, Altman DG (2009). Preferred reporting items for systematic reviews and meta-analyses: the PRISMA statement. Ann Intern Med.

[CR52] Murad MH, Asi N, Alsawas M, Alahdab F (2016). New evidence pyramid. Evid Based Pract.

[CR53] Nelson MA (1989). Androgenic-anabolic steroid use in adolescents. J Pediatr Health Care.

[CR54] Norris H (1971). The action of sedatives on brain stem oculomotor systems in man. Neuropharmacology.

[CR55] O’Connor DB, Archer J, Wu FW (2001). Measuring aggression: self-reports, partner reports, and responses to provoking scenarios. Aggress Behav.

[CR56] *O’Connor DB, Archer J, Hair WM, Wu FC (2002) Exogenous testosterone, aggression, and mood in eugonadal and hypogonadal men. Physiol Behav 75:557-566 10.1016/s0031-9384(02)00647-9 12062320

[CR57] *O’Connor DB, Archer J, Wu FC (2004) Effects of testosterone on mood, aggression, and sexual behavior in young men: a double-blind, placebo-controlled, cross-over study. J Clin Endocrinol Metab 89:2837-2845. 10.1210/jc.2003-031354 15181066

[CR58] Orwin RG (1983). A fail-safe N for effect size in meta-analysis. J Educ Stat.

[CR59] *Panagiotidis D, Clemens B, Habel U, Schneider F, Schneider I, Wagels L, Votinov M (2017) Exogenous testosterone in a non-social provocation paradigm potentiates anger but not behavioral aggression. Eur Neuropsychopharmacol 27:1172-1184 10.1016/j.euroneuro.2017.07.006 28939164

[CR60] Pereira E, Moyses SJ, Ignácio SA, Mendes DK, da Silva DS, Carneiro E (2019). Anabolic steroids among resistance training practitioners. PLoS One.

[CR61] Pope HG, Katz D (1987). Bodybuilder’s psychosis. Lancet.

[CR62] Pope HG, Kouri EM, Powell KF, Campbell C, Katz DL (1996). Anabolic-androgenic steroid use among 133 prisoners. Compr Psychiatry.

[CR63] *Pope Jr HG, Kouri EM, Hudson JI (2000) Effects of supraphysiologic doses of testosterone on mood and aggression in normal men: a randomized controlled trial. Arch Gen Psychiatry 57(2):133-140 10.1001/archpsyc.57.2.133 10665615

[CR64] Pope HG, Kanayama G, Verster J, Brady K, Galanter M, Conrod P (2012). Anabolic–androgenic steroids. Drug abuse and addiction in medical illness: Causes, consequences and treatment.

[CR65] Sagoe D, Pallesen S (2018). Androgen abuse epidemiology. Curr Opin Endocrinol Diabetes Obes.

[CR66] Sagoe D, Andreassen CS, Pallesen S (2014). The aetiology and trajectory of anabolic-androgenic steroid use initiation: a systematic review and synthesis of qualitative research. Subst Abuse Treat Prev Policy.

[CR67] Sagoe D, Molde H, Andreassen CS, Torsheim T, Pallesen S (2014). The global epidemiology of anabolic-androgenic steroid use: a meta-analysis and meta-regression analysis. Ann Epidemiol.

[CR68] Sagoe D, McVeigh J, Bjørnebekk A, Essilfie MS, Andreassen CS, Pallesen S (2015). Polypharmacy among anabolic-androgenic steroid users: a descriptive metasynthesis. Subs Abuse Treat Prev Policy.

[CR69] Salinas M, Floodgate W, Ralphs R (2019). Polydrug use and polydrug markets amongst image and performance enhancing drug users: implications for harm reduction interventions and drug policy. Int J Drug Policy.

[CR70] Schneider F, Gur RC, Gur RE, Muenz LR (1994). Standardized mood induction with happy and sad facial expressions. Psychiatry Res.

[CR71] Siegel JM (1986). The Multidimensional Anger Inventory. J Pers Soc Psychol.

[CR72] Smit DL, Buijs MM, de Hon O, den Heijer M, de Ronde W (2020a) Positive and negative side effects of androgen abuse. The HAARLEM study: a one-year prospective cohort study in 100 men. Scand J Med Sci Sports 31(4). 10.1111/sms.13843 10.1111/sms.13843 33038020

[CR73] Smit DL, de Hon O, Venhuis BJ, den Heijer M, de Ronde W (2020). Baseline characteristics of the HAARLEM study: 100 male amateur athletes using anabolic androgenic steroids. Scand J Med Sci Sports.

[CR74] *Su TP, Pagliaro M, Schmidt PJ, Pickar D, Wolkowitz O, Rubinow DR (1993) Neuropsychiatric effects of anabolic steroids in male normal volunteers. JAMA 269:2760-2764 8492402

[CR75] Taylor WN (1987). Synthetic anabolic-androgenic steroids: a plea for controlled substance status. Phys Sportsmed.

[CR76] Thiblin I, Garmo H, Garle M, Holmberg L, Byberg L, Michaëlsson K, Gedeborg R (2015). Anabolic steroids and cardiovascular risk: a national population-based cohort study. Drug Alcohol Depend.

[CR77] Tragger J (1988). Beware “roid rage” in athletes. Med Tribune.

[CR78] *Tricker R, Casaburi R, Storer TW, Clevenger B, Berman N, Shirazi A, Bhasin S (1996) The effects of supraphysiological doses of testosterone on angry behavior in healthy eugonadal men--a clinical research center study. J Clinical Endocrinol Metab 81:3754-3758 10.1210/jcem.81.10.8855834 8855834

[CR79] Wagels L, Votinov M, Kellermann T, Eisert A, Beyer C, Habel U (2018). Exogenous testosterone enhances the reactivity to social provocation in males. Front Behav Neurosci.

[CR80] *Yates WR, Perry PJ, MacIndoe J, Holman T, Ellingrod V (1999) Psychosexual effects of three doses of testosterone cycling in normal men. Biol Psychiatry 45:254-260 10.1016/s0006-3223(98)00028-6 10023498

